# Utility of abdominal skin plus subcutaneous fat and rectal mucosal biopsy in the diagnosis of AL amyloidosis with renal involvement

**DOI:** 10.1371/journal.pone.0185078

**Published:** 2017-09-19

**Authors:** Ting Li, Xianghua Huang, Shuiqin Cheng, Liang Zhao, Guisheng Ren, Wencui Chen, Qingwen Wang, Caihong Zeng, Zhihong Liu

**Affiliations:** 1 National Clinical Research Center of Kidney Disease, Jinling Hospital, Medical School of Southeast University, Nanjing, China; 2 National Clinical Research Center of Kidney Diseases, Jinling Hospital, Nanjing University School of Medicine, Nanjing, China; Kermanshah University of Medical Sciences, ISLAMIC REPUBLIC OF IRAN

## Abstract

**Objectives:**

Skin fat biopsy of the abdominal wall is a simple and safe method for detecting amyloidosis, and rectal mucosal biopsy is also frequently used for screening for the disease; however, the sensitivity of these approaches has not been fully studied. The aim of this study was to evaluate the efficacy of skin fat biopsy combined with rectal mucosal biopsy as a screening procedure for the diagnosis of systemic immunoglobulin light-chain (AL) amyloidosis.

**Methods:**

We retrospectively analyzed 224 AL amyloidosis patients confirmed by renal biopsy, including a test group of 165 patients and validation group of 59 patients. Surgical skin fat biopsy from the abdominal wall and rectal mucosal biopsy under endoscopy was performed to obtain specimens. Congo red staining and immunofluorescence staining with antibodies against light chains were performed to type the disease. Pathology reports were reviewed to assess the diagnostic sensitivity of skin fat biopsy and rectal mucosal biopsy. Diagnostic specificity was not examined in the present study, because no healthy volunteers and only few patients with other diseases had performed immunofluorescence staining on skin fat and rectal specimens.

**Results:**

Of the 165 patients in the test group, Congo red staining of skin fat and rectal mucosal specimens was associated with a sensitivity of 89.3% and 94.8%, respectively. The sensitivity increased to 98.9% by combining both biopsy methods. Immunofluorescence stains were positive in 81.1% of patients undergoing skin fat biopsy and 84.7% of patients undergoing rectal mucosal biopsy. Immunofluorescence stains yielded positive results in 86.7% of cases combining skin fat biopsy with rectal mucosal biopsy. The diagnostic results also performed well in the validation group.

**Conclusions:**

Surgical skin biopsy including the subcutaneous fat pad can be performed safely at the bedside and is useful for diagnosing AL amyloidosis. Combining skin fat biopsy with rectal mucosal biopsy may identify amyloid deposits in almost all patients, and a negative result of both biopsies makes the diagnosis very unlikely.

## Introduction

AL amyloidosis, caused by the extracellular deposition of fibril-forming monoclonal immunoglobulin light chains secreted from neoplastic plasma cells or B cell clones, is the most common type of systemic amyloidosis, with an incidence of 8.9 per million person-years [1, 2]. Patients with AL amyloidosis have a poor prognosis, with a median overall survival time of 1–2 years in untreated individuals [3]. Early and accurate confirmation and typing of amyloidosis are the keys to effective management.

The diagnosis and classification are based on histological demonstration of amyloid deposits and identification of the amyloid precursor protein [4]. Biopsies of the involved visceral organs have a high sensitivity for detecting amyloid deposition; however, such invasive procedures may carry significant risks, including hemorrhage and arrhythmia [5]. Biopsies of superficial tissues, such as the rectum, labial salivary glands, skin, abdominal fat, and bone marrow, are preferred for diagnosis as relatively less invasive procedures [6–8]. Subcutaneous fat aspiration (SFA) is a safe, simple, and low-cost method for obtaining tissue to diagnose systemic amyloidosis with a sensitivity of 67–93% [8–10]. However, patients in the early stages of amyloidosis have scant amyloid deposits, which significantly reduce the sensitivity of Congo red staining [11]. To obtain adequate tissue samples for accurate diagnosis and classification, surgical skin biopsy including the subcutaneous fat pad has been applied in our center. It can be performed safely at the bedside with high sensitivity.

Rectal biopsy was considered the gold standard in screening for AL amyloidosis before the introduction of SFA. Its sensitivity ranges from 75% to 80% [8, 12]. Combining the superficial tissue examinations shows a higher diagnostic sensitivity. Gertz et al. have reported that combining abdominal fat aspiration with bone marrow biopsy yields a detection rate of 85% for diagnosing AL amyloidosis [5]. However, the diagnostic sensitivity of combining skin fat biopsy with rectal mucosal biopsy has not been studied. The aim of this study was to evaluate the diagnostic sensitivity of skin fat biopsy combined with rectal mucosal biopsy for diagnosing AL amyloidosis, by comparing renal biopsy in the same patient population.

## Patients and methods

### Patients

This retrospective study included two groups of Chinese patients with AL amyloidosis to study the diagnostic sensitivity of skin fat and rectal mucosal biopsy. The first group comprised 165 patients who were diagnosed via renal biopsy at our institution between December 2008 and December 2015. The second group, referred to as the validation group, comprised 59 consecutive patients undergoing renal biopsy at other institutions during the same period. The renal specimens were submitted to our institution for further examination; the skin fat and/or rectal mucosal biopsies were also performed in this cohort. The study protocol was approved by the institutional ethical review board of Jinling Hospital, and written informed consent was obtained from each participant to have their medical records reviewed.

The diagnosis of AL amyloidosis was confirmed by renal biopsy, which was made histologically by Congo red stain and confirmed by immunofluorescence staining for the expression of kappa or lambda light chains. The diagnosis was also supported by documented plasma cell dyscrasia (serum M protein or an abnormal free light chain ratio). In addition to renal biopsy, the majority of patients also underwent surgical abdominal skin and subcutaneous fat biopsy and rectal mucosal biopsy, and some patients underwent three biopsies. Renal, skin fat and rectal mucosal samples were all obtained during the first hospitalization and were evaluated by different pathologists who were blinded to the patient clinical information and other pathological findings. The assessment of organ involvement was based on consensus criteria [13]. The revised Mayo staging system was used to assess the disease stage of all the patients [14]. Demographic, clinical and laboratory data were obtained from electronic medical records and used for the analysis. All patients underwent bone marrow cytology, and bone marrow biopsies were examined for Congo red staining in 86 patients. Patients who were diagnosed in the same period were excluded because they had received previous chemotherapy and clinically overt multiple myeloma. No patient with senile, secondary, familial, or localized (including dialysis-related) amyloidosis was included.

### Histopathological study

Ultrasound-guided renal biopsy was performed in all patients. Renal biopsy samples were examined by routine light microscopy, immunofluorescence (IF) and electron microscopy (EM). Amyloid deposition was determined by the presence of salmon-colored Congo red deposits with apple-green birefringence under polarized light. By EM, amyloid appears as randomly arrayed non-branching fibrils with diameters of 8–12 nm. The subtype of amyloid was determined by IF stains for the expression of kappa or lambda light chain. For all renal biopsies, the sections were treated with potassium permanganate before Congo red staining and stained with the monoclonal anti-human AA antibody. Furthermore, in cases with equivocal light chain expression, additional immunofluorescence studies were performed to exclude hereditary amyloidosis (i.e., apolipoprotein A, fibrinogen, leukocyte chemotactic factor 2, lysozyme, and transthyretin).

Surgical biopsy of abdominal wall skin and subcutaneous fat was performed in 172 patients (121 in the test group and 51 in the validation group); the skin sample was approximately 2–4 mm in width including dermal tissue, and subcutaneous fat of the same size was removed. The incision was sutured with non-absorbable silk. Rectal mucosal biopsy under endoscopic control was performed in 188 (134 in the test group and 54 in the validation group) patients. The formalin-fixed and paraffin-embedded skin fat and rectal sections were stained with Congo red for the diagnosis of amyloidosis, and indirect IF staining was performed on the same tissues to determine the amyloid subtype.

### Statistical analysis

Categorical variables are expressed as absolute numbers and percentages. Continuous variables are expressed as the median (interquartile range) according to the skewed distribution. The Chi-square test was used to test differences in categorical variables. Differences in continuous variables between groups were compared using the Mann-Whitney U test. A probability (p) value of 0.05 was considered significant for all tests. All statistical analyses were performed using SPSS version 18.0 (IBM Corp., Armonk, NY, USA).

## Results

### Patients’ characteristics at biopsy

For all 224 patients, the median age was 56 (range: 30–75) years, and 65.6% of them were male. All patients had different degrees of proteinuria, with a median level of 4.28 g/24 h (interquartile range: 2.42–6.71 g/24 h). Nephrotic syndrome was present in 46.4% (104) of the patients, and 29.9% (67) of patients presented with impaired kidney function (eGFR<90 ml/min per 1.73 m2) at the time of diagnosis. After the kidney, the most commonly involved visceral organ was the heart (45.5%). The median levels of N-terminal B-type natriuretic peptide (NT-proBNP) and interventricular septum (IVS) were 464.1 pg/ml (interquartile range: 154.8–1682.9 pg/ml) and 11 mm (interquartile range: 10–13 mm), respectively. We divided all the patients into four subgroups according to the Mayo staging system. The proportions of patients with stages I, II, III and IV were 70 (31.2%), 66 (29.5%), 63 (28.1%) and 25 (11.2%), respectively. In addition to the kidney and heart, intestinal and soft tissues were also commonly involved. The highly involved rate of intestinal and soft tissues may be due to the selection of the puncture site. In terms of hematologic parameters, the κ/λ FLC ratio was abnormal (<0.26 or >1.65) in 53.1% of patients, and M protein was identified in 70.0% of patients using immunofixation electrophoresis (IFE). Combining the serum free light chain (FLC) analysis and IFE, monoclonal FLC was detected in 75.5% of patients. The demographic and clinical characteristics of 224 patients are listed according to study group in Table 1. The two groups were similar with respect to sex (p = 0.2), heart involvement (p = 0.2), serum M-spike (p = 0.4). The age (56 vs 54 years, p = 0.04) was older in the test group than in the validation patients.

**Table 1:** Baseline characteristics of 224 patients diagnosed with AL amyloidosis via renal biopsy.

	Test Cohort (n = 165)		Validation Cohort (n = 59)	
Characteristic	No.	%	No.	%
Median age (years)	56		54	
25th to 75th centile	49–63		47–59	
Sex	54.5	53.1	50.7	53.9
Male	104	63.0	43	72.9
Female	61	37.0	16	27.1
Organ involvement at diagnosis				
Renal	165	100.0	59	100.0
Cardiac	71	43.0	31	52.5
Intestinal tract	99	60.0	44	74.6
Soft tissue	110	66.7	49	83.1
Liver	19	11.5	5	8.5
Peripheral nerve	10	6.1	3	5.1
Mayo cardiac stage (2012)				
I	54	32.7	16	27.1
II	49	29.7	17	28.8
III	44	26.7	19	32.2
IV	18	10.9	7	11.9
Biopsy sites				
Renal	165	100.0	59	100.0
Skin/fat	121	73.3	51	86.4
Rectal mucosal	134	81.2	54	91.5
Performing all biopsies	90	54.5	46	78.0
Median BMPC (%)	2.0		2.0	
25th to 75th centile	(1.0–5.0)		(1.0–4.0)	

Abbreviation: BMPC, bone marrow plasma cells.

### Pathologic results

**Fig 1:**
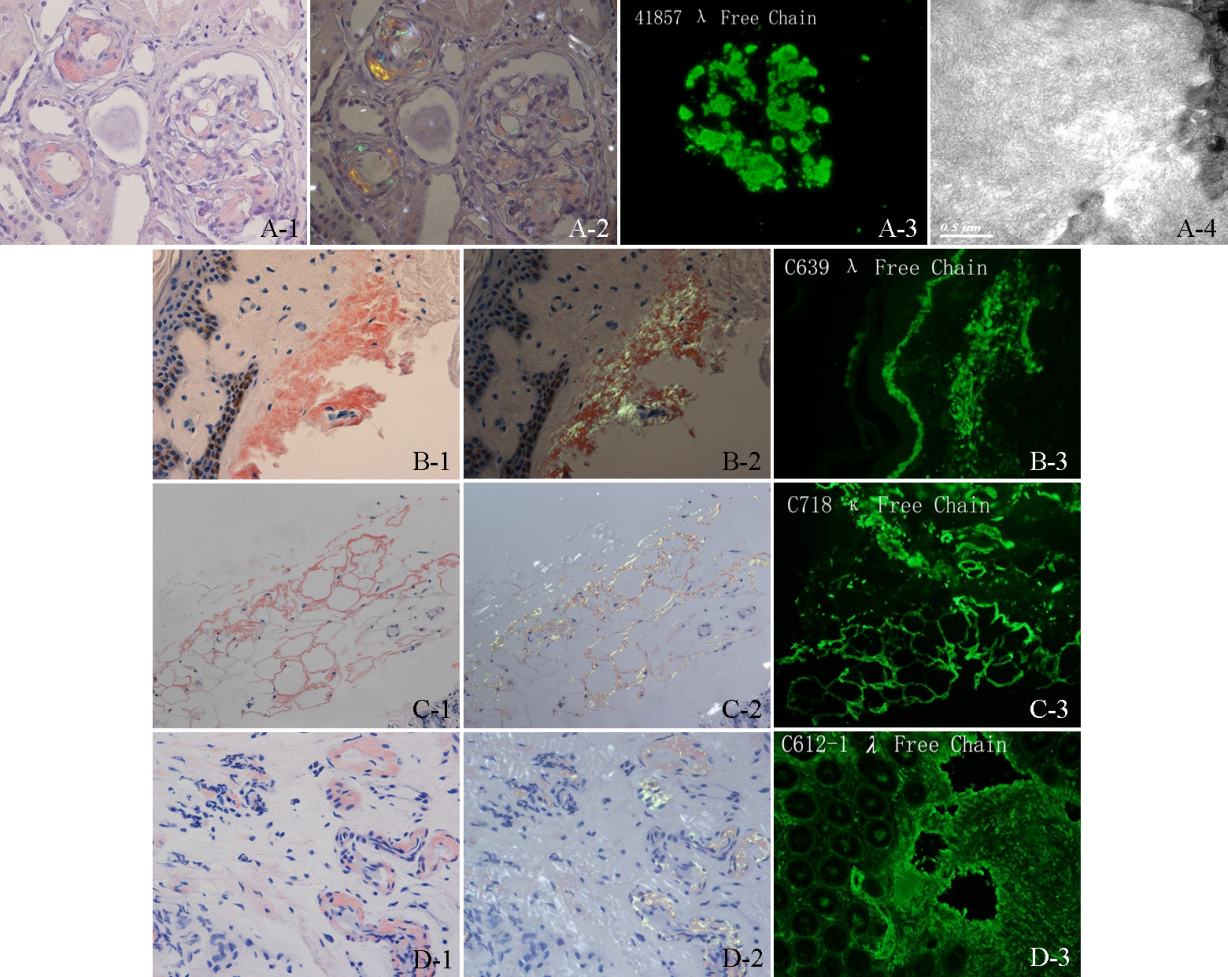
Typical staining of amyloid deposits in patients with AL amyloidosis. Amyloid deposits seen in renal, skin, fat, and rectal mucosal specimens (i.e. Congo red staining, immunofluorescence staining, and electron microscopy). (A-1) Renal specimen show amyloid deposits in mesangial, capillary loop, and the wall of small artery (Congo red, x 400). (A-2) Amyloid deposits show apple green birefringence under polarized light of renal tissue (Congo red, x 400). (A-3) Monoclonal staining with lambda light chain in mesangial (immunofluorescence, x 400). (A-4) Electron micrograph of straight, non-branching, randomly organized 8–10 nm diameter fibrils in mesangial (B-1) Skin specimen show amyloid deposits in dermis (Congo red, x 400). (B-2) Apple green birefringence under polarized light in dermis (Congo red, x 400). (B-3) Monoclonal staining with lambda light chain in dermis (immunofluorescence, x 400). (C-1) Amyloid deposits surround individual adipocytes (Congo red, x 200). (C-2) Apple green birefringence under polarized light surround fat cells (Congo red, x 200). (C-3) Monoclonal staining with kappa light chain in fat tissue (immunofluorescence, x 200). (D-1) Amyloid deposits in the wall of small blood vessels in rectal mucosal tissue (Congo red, x 400). (D-2) Apple green birefringence under polarized light in the wall of small blood vessels (Congo red, x 400). (D-3) Monoclonal staining with lambda light chain in the submucosa layer (immunofluorescence, x 200).

The Congo red staining results in 224 patients with AL amyloidosis are displayed by subgroup in Table 2. The typical histological pictures are shown in Fig 1. We first examined the Congo red results in the test subgroup. Of the 165 patients, 121 patients received skin fat biopsy, 108 (89.3%) of which were positive, and 134 patients received rectal biopsy, with a positive rate of 94.8% (127). Among 90 patients combining both skin fat and rectal mucosal examinations, only one patient was Congo red negative in both specimens, with a diagnostic sensitivity of 98.9%. Among the 59 patients in the validation subgroup, the two methods also performed well; combining both methods, all of them had at least one biopsy sample stained positive in Congo red staining. Taking the two groups together, 86 patients had their bone marrow specimens stained with Congo red, whereas only 22.1% (19) were positive.

**Table 2:** Congo red staining results in patients with AL amyloidosis.

Biopsy sites	No.	Sensitivity (%)	95% CI
Test cohort (n = 165)			
Skin/fat (n = 121)	108	89.3	83.5–94.2
Rectal mucosal (n = 134)	127	94.8	91.0–98.5
Either skin/fat or rectal mucosal (n = 90)	89	98.9	96.7–100.0
Validation cohort (n = 59)			
Skin/fat (n = 51)	47	92.2	84.3–98.0
Rectal mucosal (n = 54)	51	94.4	87–100.0
Either skin/fat or rectal mucosal (n = 46)	46	100.0	——

We further examined the amyloid deposit site in all 224 patients (Table 3). Among the 155 skin fat positive patients, the subcutaneous tissue was the most common site of amyloid deposition (91 patients, 58.7%), followed by the dermis in 45.8% (71 patients) and the blood vessels in 36.1% (56 patients). In the rectal positive patients, the blood vessel was the most common site of amyloid deposition (112/178, 62.9%), followed by the mucosal layer (76/178, 42.7%) and the submucosa layer (72/178, 40.5%).

**Table 3:** Deposit sites of amyloid in skin fat and rectal mucosal.

Biopsy sites	No.	Sensitivity (%)	95% CI
Skin/fat (n = 155)			
Dermis	71	45.8	38.1–53.5
Subcutaneous tissue	91	58.7	50.3–66.5
Blood vessel	56	36.1	28.4–43.2
Rectal mucosal (n = 178)			
Mucosal layer	76	42.7	34.8–50.0
Submucosa layer	72	40.5	33.7–47.8
Blood vessel	112	62.9	55.1–70.2

The immunofluorescence results are displayed by subgroup in Table 4. The 165 patients in the test group were further categorized into AL amyloid of κ-light chain origin [22 (13.3%) patients] or λ-light chain origin [143 (86.7%) patients] using immunofluorescence. Among them, immunofluorescence revealed positive staining of monoclonal light chains in 81.1% (30/37) of the skin fat specimens and in 84.7% (50/59) of the rectal specimens. Combining the two examinations caused an increase in the classification rate to 86.7 (13/15). Amyloid remained unclassified for the following reasons: (a) None of the antibodies immunostained the amyloid deposits in 1 patient, who showed λ-free light chain deposit in renal specimens, and the corresponding circulation involved monoclonal light chain in serum analyses. (b) One patient's Congo red stain was negative in both skin fat and rectal specimens, which were double negative on immunofluorescence staining. In this patient, immunostaining of the renal specimen revealed positive staining of the κ-free light chain, and the serum analyses also revealed an abnormal free light chain ratio (κ: 140.59 mg/L; λ: 6.59 mg/L; κ/λ: 21.33) and M protein (IgGκ). In the validation group, patients with AL amyloid of κ-light chain origin and λ-light chain origin numbered 6 (10.2%) and 53 (89.8%), respectively. The numbers of patients who underwent skin fat and rectal mucosal immunofluorescence staining in the validation group were 10 and 37, respectively. The classification rates were 80.0% and 91.9%. Combining results from the assay of skin fat and rectal specimens, immunofluorescence yielded positive staining for all 7 patients.

**Table 4:** Immunofluorescence results in patients with AL amyloidosis.

	Test cohort (n = 165)		Validation cohort (n = 59)	
Biopsy sites	No.	Sensitivity (95% CI)	No.	Sensitivity (95% CI)
Skin/fat	30/37	81.1 (67.6–91.9)	8/10	80.0
Kappa	3		0	
Lambda	27		8	
Difficult to determine	7		2	
Rectal mucosal	50/59	84.7 (74.6–93.2)	34/37	91.9 (83.8–100.0)
Kappa	3		4	
Lambda	47		30	
Difficult to determine	9		3	
Either skin/fat or rectal mucosal	13/15	86.7	7/7	100.0
Kappa	2		1	
Lambda	11		6	
Difficult to determine	2		0	

## Discussion

Tissue biopsy is the gold standard for the diagnosis and typing of amyloidosis. The biopsy of involved organs yields high diagnostic sensitivity and specificity; however, the biopsy of a clinically suspected organ is an invasive procedure and may be associated with complications including hemorrhage [5]. SFA, rectal, skin, labial salivary gland, and bone marrow biopsies are frequently used to detect amyloid deposits in patients with symptoms of the disease. However, the sensitivity values of these approaches vary greatly, and the values in Chinese patients remain to be elucidated. In our previous study, we confirmed the sensitivity of rectal mucosal biopsy to be 86.7%, and the analysis of abdominal wall skin fat specimens, obtained by surgical procedures, yielded a sensitivity of 92.5% [15]. In the current study, we thoroughly evaluated the efficiency of skin fat and rectal biopsy on the diagnosis of AL amyloidosis in a cohort of patients with renal involvement. By examining biopsy samples of skin fat and rectal mucosal, we detected amyloid deposits in 89.3% and 94.8% of patients, respectively. Combining both methods, only one patient had a negative result, which suggests that skin fat and rectal biopsies are appropriate superficial biopsy sites for diagnosing AL amyloidosis.

Currently, SFA is one of the widely used methods for screening and diagnosing systemic amyloidosis, which has a high specificity of 92%-100%. According to the literature, the sensitivity range is wide, from 67% to 93% [8–10], for which Ingrid I. van Gameren et al. reported the usefulness of a thorough assessment (3 fat smears, 2 observers), with an increased sensitivity from 80% to 93% [10]. In this study, we observed a sensitivity of 89.3%-92.2% for skin fat biopsy, which is similar to the best result reported in the literature. The main reasons for the high sensitivity of our study may include the following: 1. Surgical skin biopsy including the subcutaneous fat pad can provide an adequate amount of tissue for analysis and increase the sensitivity. In some patients, positive staining was observed exclusively in skin samples and vice versa in fat tissues, which proved the possibility. 2. The differences in the study population and the number of research subjects may also affect the results. The results of this study were based on a large cohort of patients, making the results more reliable. 3. All of the patients in this study had renal involvement, and nearly half of the patients had concurrent heart involvement. Studies have suggested that renal involvement is associated with a higher prevalence of λ light-chain amyloid [16]. It is undetermined whether the type and number of involved organs and the type of light chain affect the positive rate of skin biopsy.

Rectal biopsy has been the commonly used procedure for diagnosing amyloidosis since its introduction in the 1960s, with a sensitivity of 75%-80% [8, 12]. In this study, we observed a relatively higher positive staining rate of rectal biopsy than that reported in the literature. In our comparison of samples from the same patient population, the sensitivity of rectal biopsy was higher than the skin biopsy. In addition to the differences in the study population and the number of research subjects, technological advances in biopsy and pathological analyses may be the main reason. We observed that 40.5% of amyloid substances were deposited in the submucosa layer. Therefore, for the effective diagnosis of AL amyloidosis, the rectal specimens should include the submucosa. Combining applications of the superficial tissue biopsies yielded a higher diagnostic sensitivity. In this study, only one patient was Congo red negative in both skin and rectal biopsies. The results indicate that excellent diagnostic sensitivity can be obtained by combining the two methods.

Correct classification of the amyloid is of paramount importance because treatment differs in accordance with the type of amyloidosis. Immunofluorescence and immunohistochemistry are frequently used, and the former is more accurate for AL typing because of the lower background staining in this method [17]. Immunogold electron microscopy can also be used for diagnosis but is mainly used for research. Although the number of patients was small, we still observed relatively good diagnostic sensitivity of immunofluorescence staining in skin fat biopsies and rectal mucosal biopsies. Combining the two biopsy methods, there were two patients showing negative results in staining with antibodies against both κ and λ FLC. The main reason for negative results might be rooted in the following: 1. The constant domain of light chain in amyloid deposits may be partial or complete deletion due to local proteolysis, result in non-recognizing of commercial antibodies. 2. Because of the genetic mutations and conformational changes of amyloid precursors, the commercial antibodies may be non-reactive with the mutant form. 3. Insufficient amounts of samples are obtained for accurate diagnosis, leading to false-negative results. 4. No amyloid deposits are present in the biopsy tissues. In addition, we observed staining of the same amyloid deposit with both anti-λ-light chain and anti-κ-light chain antibodies. This may be related to the contamination of amyloid by serum proteins. Thus, negative or equivocal staining for immunoglobulin light chains does not automatically rule out AL. Although it is currently used only in specialized centers, laser microdissection followed by mass spectrometry (LMD-MS) is the gold standard for amyloid typing [18]. Mass spectrometry (MS) has been applied for typing amyloidosis using fat aspirates [19, 20]. The classification efficacy of MS should be validated in skin fat and rectal biopsies, based on the good performance of the biopsies in Congo red staining.

Approximately 70% of AL patients have complications with renal involvement, which is the most common presentation in this condition [21]. Renal biopsy has high specificity and sensitivity in identifying amyloid depositions [8]. Moreover, it is necessary for the diagnosis as combined renal disease is suspected [22]. In total, 165 patients in this study underwent renal biopsy in our center, and no serious bleeding or other severe complications were observed. No increased incidence of complications was found in AL patients who underwent renal biopsy, compared with the other patients. Therefore, a renal biopsy should be performed if combined renal disease is suspected or the suspicion index of AL is high without positive results in skin fat and rectal biopsies.

This study has several noteworthy limitations. First, all of the patients were diagnosed by renal biopsy, which might have resulted in a selection bias. Because we did not investigated patients without renal involvement, in whom the amyloid depositions were confirmed by examining other organs or surrogate samples, the diagnostic efficiency of the skin fat and rectal mucosal biopsy might be relatively different in this group of patients. Second, MS analysis is currently regarded as the gold standard for typing amyloidosis. However, the technology has not been applied in our center until recently. Hence, the amyloid type was determined by immunofluorescence staining in this study. Third, the relatively small number of patients who underwent immunofluorescence staining using skin fat and rectal mucosal samples possibly resulted in the inaccurate assessment of the typing sensitivity. Finally, given the high specificity of the Congo red staining for diagnosing AL amyloidosis, we did not perform specificity analysis in the present study. The specificity assessment was not performed for immunofluorescence staining due to the small sample size of control groups. The above-mentioned factors may influence the extrapolation of these results to the clinical practice.

In summary, skin fat and rectal mucosal biopsies are safe and simple procedures with a high diagnostic sensitivity for AL amyloidosis. Combined biopsies of both sites should be performed to obtain more-reliable diagnostic results and a higher sensitivity. If the results are negative, the likelihood of AL amyloidosis is low. Due to the lack of a specificity study, the reliability of immunofluorescence amyloid typing on skin fat and rectal mucosal specimens remains to be further validated.
