# Correction: Utility of abdominal skin plus subcutaneous fat and rectal mucosal biopsy in the diagnosis of AL amyloidosis with renal involvement

**DOI:** 10.1371/journal.pone.0187909

**Published:** 2017-11-06

**Authors:** The PLOS ONE Staff

There is an error in affiliation 1 for author Ting Li. Affiliation 1 should be: School of Clinical Medicine, Southeast University, Nanjing, China. Additionally, Xianghua Huang and Zhihong Liu should also be affiliated with School of Clinical Medicine, Southeast University, Nanjing, China.

The following information is missing from the Funding section: This work was supported by grants from the Jiangsu Provincial Medical Youth Talent (QNRC2016895), and Key Research and Development Plan Project of Jiangsu Province—Social Development Projects (BE2017721). The publisher apologizes for this error.
